# Dental Hints Department

**Published:** 1906-11

**Authors:** B. H. Teague

**Affiliations:** Aiken, S. C.


### DENTAL HINTS DEPARTMENT

**Conducted by B. H. TEAGUE, D. D. S., Aiken, S. C., to whom all contributions to this department should be sent.**

It would seem that sponge gold and cohesive tin, layer upon layer, would make a good filling.

An adult man passes daily from his bowels from 30 to 50 billions of bacteria, say Vignal and Suckdorf.

There is nothing that the cocaine addict will not do to get the drug. He has no shame.—*Am. Jour. Clin. Med.*

The prescription pad is the sole therapeutic armamentarium of 90 per cent of the medical profession.—*Southern Med. and Surg.*

If I were but a dentist, I'd never have to beg. The dentist pulls your wisdom teeth, and then he pulls your leg.—*Stomotologist.*

Camphor and phenol, equal parts evaporated by heat 4 oz. to 1,000 square feet, kills mosquitos and stuns houseflies.—*Am. Jour. Clin. Med.*

Every ailment would become a poison if renal elimination was not the safeguard of the body.—BOUCHARD, *Am. Jour. Clin. Med.*

Great muscular activity in the open air in the country lessens the toxicity of the urine by one-third.—BOUCHARD, *Am. Jour. Clin. Med.*

BETA-EUCAIN LACTATE.—Beta-eucain is water-soluble to 3½ per cent.; beta-eucain lactate is water-soluble to 22 per cent., and has all the properties of the hydrochlorid.—*St. Louis Med. Review.*

In taking an impression where retching is bound to supervene, making the operation almost impossible. I have succeeded by swabbing the palate with Wickoff's Obtundent.—Dr. J. E. MCDONALD, *Dom. Jour.*

Ethyl Chloride; a Warning.—Ethyl chloride should not be given in any case of laryngeal irritation, Graves' disease, in cardiac disease, or to alcoholic and neurasthenics; in fact only to healthy subjects.—W. B. Pritchard, *Dental Record.*

As to the wearing out of the filling on the grinding surface of molars where from one-half to two-thirds of the surface is exposed to wear and stress. The ten parts of gold to one of tin will stand that wear. Dr. Callahan, *Dentist.*

Sterilization of Cavities.—In the sterilization of cavities, preparatory to filling, phenol solique is unsurpassed. It does not injure the dentin, and being employed undiluted is prompt in action and always ready for immediate use.

Exposure of cement liquid to the moist atmosphere of summer time results in the taking up of water. Exposure to the dry atmosphere of average office in midwinter results in the giving up of water.—*Summary.*

Palatal nodules in the upper arch are always to be viewed with gravity; and careful examination and study should be made of the topography of the parts so as to give relief to the hard places, thus equalizing the pressure with the soft places in the roof.—*Dental Magazine.*

Chloro-Percha.—In case of evaporation of the chloroform when wanted for immediate use, add the solvent and immerse the container in a dish of hot water. The chloroform begins to boil forthwith and the material is ready for immediate application.—*Dental Office and Laboratory.*

While our cements are as yet only temporary, their period of usefulness may be greatly extended by proper care and procedure in manipulation. It is our personal belief that the life of a cement filling is affected more by the operator's manipulation than by the oral secretions of the patient.—*Summary.*

When you find a flap of gum tissue, grown over and into an approximal cavity, try ligaturing it to the adjacent tooth with waxed silk floss. This will cut off the circulation by strangulation and the gum flap may be removed without pain, using the lancet and applying adrenalin or adnephrin to arrest hemorrhage.—Dr. J. E. McDonald, *Dom. Journal.*

Charged With Larceny.—Mrs. Anna Cohn, wife of a junk dealer, caused a warrant to be issued for the arrest of Dr. Davis, a dentist at Freeport, Ill., charging him with appropriating to his own use gold that was contained in two crowns she left at his establishment. Davis was arrested and lodged in the county jail.—*Amer. Dental Journal.*

Backing for Facings.—A very satisfactory backing for facings is obtained by using 1-1000 platinum, doubled and burnished to the facing. Its advantages are: Adaptability to the facing, less liklihood of burning the backing while soldering, and reflection of color through facing.—Henry C. Lee, *Dental Review.*

It is also necessarily true that the heavier the solution the greater will be the tendency to crystallization. Thus we see that addition of water must quicken setting, and abstraction of water must retard setting.—*Summary.*

Cement should be mixed to different consistencies for various purposes.

Heat and cold, dry and humid atmospheric conditions have a marked effect on the cement.

The neck of the cement liquid bottle should not be left wet, as is usually the case.—*Summary.*

On opening up a pulp-chamber in which there is a putrescent pulp, giving out a particularly offensive odor, dip your broach in oil of turpentine and insert in canal, the odor will change most agreeably, both to yourself and your patient, almost instantly.—Dr. J. E. McDonald, *Dom. Jour.*

To Prepare the Floor of the Cap for a Porcelain Crown.—Puncture the floor of the cap from the root side with a pointed three-cornered instrument. A puncture thus made turns up a bur on the porcelain side, which becomes firmly embedded in the body of the piece, and is fully as retentive as a headed pin in the facing.—L. A. Arnold, *Dental Review.*

School of Anesthesia and Extraction of Teeth.—The only school of anesthesia and extraction existing has been started in Chicago for thorough instruction in this branch of dentistry. Dr. L. W. Nevius is dean; Dr. L. O. Green, president; Dr. O. W. Green, consulting physician, and Dr. F. K. Ream, secretary. There are said to be good opportunities in the cities for specialists in this branch.—*Summary.*

THE ADMINISTRATION OF ANAESTHETICS.—No man has the right to operate and administer an anæsthetic at the same time. —DR. DARBY.

The highest courts in the land have not passed upon the province of the dentist to administer anesthetics; and some time that will take place; and when it does it is to be hoped we shall be represented by some one who can do credit to himself and the profession of dentistry.—Dr. C. P. PRUGN, *Review*.

RETENTION GROOVE FOR MATRICES.—Use thin copper plate for inlay matrix. Place a fold of small copper wire in bottom of matrix and run up with gold solder, using plenty of flux. Just before setting drop the inlay into nitric acid to remove the copper. The removal of the copper wire leaves a good retaining groove.—J. B. NEWELL, *Dental Review*.

LAC EDGE OF INLAY.—In inlay work paint the peripheral edges of the matrix with shellac before placing the body. The shellac will burn out and prevent warping of the inlay. The crevice is filled at the second placing of body.—*Western Dental Journal*.

THYMOL IN THE TREATMENT OF ABSCESSES.—Thymol is very insoluble under ordinary circumstances, but it dissolves in oil of eucalyptus, when it becomes a valuable agent in the treatment, especially of the mild forms of chronic blind abscesses.—GEO. W. COOK, *Western Dental Journal*.

PULP REMOVED.—The important point to remember in opening up a cavity in a tooth containing a dead pulp, is the danger of forcing infected pulp detritus through the end of the root; consequently it is important to bathe the interior of the pulp-chamber with a 10 per cent. solution of formaldehyde before instrumentation is begun.—M. L. RHEIN, *Dental Digest*.

To polish gold crowns, etc., I run my felt wheel over a cake of "lava soap." You will need very little pumice, as I think the base of it is pumice or volcanic dust. It's par-excellence for above use.—*Kansas City Dental Journal*.

TREATMENT OF VERY SORE TEETH.—I have had great help from the use of a string tied around the tooth, instructing the patient to draw on it until it is real tight. The effect will be a revelation.—F. MILTON SMITH, *International Dental Journal*.

Protection for Facings.—To prevent "etching" of facings when using 20 per cent. platinum solder, paint the surface with a creamy solution of magnesium carbonate before investing.—A. E. Matteson, *Dental Review.*

If an accident should happen in the case of a physician he is granted the privilege of signing a death certificate reading "death by shock." The dentist cannot do this; he must call in a coroner, which gives publicity, and he cannot afford to take that risk.—Dr. Bartlett, *Dental Era.*

Solid Gold Inlay.—By the use of pure gold and platinum matrix, making a solid gold inlay, all future trouble of this nature is entirely eliminated, and the result is a surface which will endure even better than that of any malletted filling. As a matter of fact, no filling is so well condensed as is the pure gold inlay.—*Summary.*

To best open the subject, we will assume that it is conceded that there is a certain desirable consistency to which an oxyphosphate of zinc cement should be mixed for filling purposes, and another consistency most desirable for the setting of crowns, bridges and inlays. There is a certain homogeneous condition (stiff) best for filling, and for crowns and inlays cement should be as stiff as will admit of the piece going readily to place.—*Summary.*

Burnishing Fillings.—In burnishing gold fillings, anchored with cement, it is better to keep the instrument warm—the heat of the instrument has a tendency to hasten the hardening of the cement so that you seldom wait for that. * * * Burnishing gold on the cement allows you to work on walls that you would not think of working on with a mallet, and you can work with much less room than you can with a mallet. The ease to a patient is a very important factor.—W. I. Brigham, *International Dental Journal.*

A Possible Danger With Ethyl Chlorid.—Dr. Amoedo of Paris says that one of his operating assistants having anesthetised the mucous membrane around the upper incisors in a young girl, for the purpose of opening a dental abcess with the thermo-cautery, the ethyl chlorid, on coming in contact with the heated instrument, became ignited. A long flame proceeded from the mouth and set fire to the eyelashes and hair of the patient.—*Digest.*

CASTS FOR CLASPS.—A method which I use in partial gold cases is, get an accurate impression with plaster of Paris and when the model is being cast pour soft plaster into the teeth and put a flat pin into them, allow it to harden, and then pour the balance of the model. When it is hard the teeth may be readily removed from the model and the plate struck up and the teeth put back in position and the clasps fitted to them, so the adjustment is absolutely accurate.—DR. STODDARD, *International Dental Journal.*

WARPING OF VULCANITE.—Plaster of Paris is capable of being compressed. Enormous pressure, far more than is necessary to compress plaster, can be produced by the appliances used for closing flasks. Vulcanizable rubber, when heated to 212 degrees F., does not flow rapidly. Therefore, if an excess of rubber is present and the case is closed rapidly under heavy pressure, distortion of some portion of the matrix will very likely occur..—J. H. PROTHERO, *Dental Magazine.*

The continued use of cocaine does not cause that "familiarity which breeds contempt," but rather a wholesome respect for and fear of such a dangerous drug. In recapitulation allow me to sound a big note of warning: (1st) Do not become reckless and careless in the use of anesthetics; (2d) do not use any preparation of drugs to produce local or general anesthesia that you are not familiar with; (3d) also do not use any of the coal tar headache cure preparations or sedative that you are not familiar with, as they are dangerous, dangerous, dangerous!—DR. C. P. PRUYN, *Review.*

A WOODEN LEG.—A suit over who shall pay for a wooden leg is in progress in Pittsburg. The question is as to whether a man who marries a girl with a wooden limb, marries the limb too. Dr. Clarence Gukert, a dentist, has been sued for $100, the cost of a wooden limb, by Dr. R. H. M. McKenzie at Pittsburg. McKenzie brought suit before an alderman, alleging that the limb was furnished some years ago to a young, woman, since Mrs. Gukert, and he wants the husband to pay for the limb. Gukert's defense was that a wooden limb was not part of any person, any more than is a shoe or a finger ring; that he should not be forced to pay for anything bought by his wife before their wedding. The alderman gave judgment for McKenzie, but Gukert has appealed the case.—*American Dental Journal.*

The Porcelain Inlay.—That porcelain has a wide range of usefulness has been demonstrated; that it has its limitations has also been demonstrated. For æsthetic reasons it is indicated for cavities which are exposed to view in laughing or speaking when such cavities can be properly shaped for retention, and where they will have sufficient bulk to stand the stress to which they will be subjected, but where fillings will not be exposed to view, where space is limited, stress is great, and sufficient retention can not be secured, porcelain is contraindicated.—Dr. Jas. H. Prothero, *Northwestern Dental Journal.*

A Good Suggestion.—Dr. J. H. Graham of Lead, S. D., sends the following suggestion: Dentists, as a rule, subscribe for from three to six dental journals, and in reading the same come across many useful hints or suggestions, which we feel we would like to try should occasion present. But having no case at the time suitable to demonstrate the value of the suggestion, or from some other cause, we lay the journal aside and the matter passes out of our mind. It has been a custom with me for a number of years to keep a scrap book, and when I come across a suggestion in a dental journal that appeals to me as of value, I cut it out and paste it in the same. In this way I get what appeals to me as the meat of our dental literature, boiled down for quick and easy reference.—*Dental Digest.*

A New and Valuable Method of Making Matrices.—I recently received from Dr. Emil Schreier, of Vienna, a suggestion which, after trial, I unhesitatingly pronounce to be one of exceeding value in the technique of all kinds of inlay fillings. There can be no doubt but that the stability of an inlay is much enhanced by the depth to which it may be introduced into the cavity. Heretofore in very deep cavities it has been necessary to lessen the depth by strata of cement or other material. Dr. Schreier suggests the use of what is known as gold beaters'skin. This is an exceedingly thin bladder-like material which gold beaters use when beating the metal into foil to avoid tearing.—*Items.*

A very prominent man in profession says he can make more money doing other work, and therefore does not intend to give his time to oral prophylaxis. He says he is in the profession for the money that is in it. It is decidedly unprofessional to take that attitude; we are not in a business, but in a *profession,* and we should not forget this: "A professional man is one who gives his life to the pursuit of his art or calling with unselfish devotion to those whom he is to serve, using every means at his command to perfect himself, both technically and scientifically. Under this definition few qualify, but all can who will."—*Summary.*

Mat of Gold at Cervix.—Dr. Green also said that he had found that a mat of gold at the cervical margins, especially in bicuspids and molars, made not only an excellent foundation for the first layers of the filling, but that it also held the matrix from the margins to that degree that he had an abundance of material for contouring and fine-finishing of the filling. The mat should consist of several layers and extend far enough into the proximal space to fold a little gumwards.—*Dental Office and Laboratory.*

How to Remark an Inlay.—Furthermore, should decay appear around the inlay at a future time the inlay may be removed, cavity prepared, and a new matrix made, placing the old inlay therein and adding the necessary amount of gold to restore lost tissues, all of which is a strong argument for the inlay over the filling. Also many teeth can be restored which are now filled with amalgam, thus contributing to your patient's comfort and well-being. Many teeth which are now crownd can be restored to usefulness, and withal be sanitary in their surroundings by the use of the gold inlay.—*Summary.*

The problem of mixing an oxyphosphate of zinc cement is one which must be largely worked out by the individual operator. One cognizant of the proper feel of the cement beneath the spatula is in position to get good results. In a general way, some points of value can be given. Most often cement does not receive sufficient careful spatulation, yet it can be utterly ruined by over-spatulation. Too little spatulation gives a quick setting granular result, and over-spatulation gives cement which will never properly crystallize. Thus it can be seen that these features must be kept in mind, and that the operator must become familiar with the proper *feel* of the cement beneath the spatula.—*Summary.*

Making the Cap and Pin (Base of the Porcelain Crown) Easily Removable.—Having taken the impression and bite with the modeling compound in the usual manner to show the relation of the occluding teeth, with a small spatula fill the cap smoothly and evenly with either plaster or modeling compound. Soap or oil the surface of this together with the whole impression—both sides. This leaves the pin protruding for the guide. Now take a piece of German silver hollow joint-wire (such as may be obtained at any jeweler's supply house, in various sizes), one that just nicely fits over the pin, and of the same length, slip it over the pin for the impression; pour both sides of the impression attaching to the articulator in the usual manner. When plaster is thoroughly set, remove the modeling compound under dry heat. The base of the crown is then at once separable from the model, the tube insuring accuracy of position in building up the crown.—GARRET NEWKIRK, *Review.*

KEEPING RECORDS.—Many are very careless in the records they keep. Occasionally you do come in contact with a dishonest patient and may be obliged to sue to recover your fee, and then you find out that your record is kept in such a way you have absolutely no legal basis upon which to base a judgment against the debtor. For that matter, you should keep all your records very carefully, and I would suggest that you keep a large appointment book, and on that appointment book you make a record in some sort of cipher, or use abbreviations, if you please, as to just what was done for that particular individual; then I suggest that you keep a card system of record, and transfer to those cards the record from your appointment book, then you have some definite legal basis upon which to institute suit upon an account, if it should become necessary.—DR. J. E. NYMAN, *Domestic Journal.*

SOLDERING.—Take the porcelain facing that is to be used in crown or bridge, fit it on to the model, bevel the occlusal margin to a fine point, being careful not to check edge, take a piece of heavy tea lead, fit it as if for backing, take off, use as a pattern to cut out and punch holes in gold or plantinum backing; fit backing on facing, burnishing it down over tip, then catch backing in soldering pliers on the tip side of holes, work the backing off, facing and holding it in pliers paint the slightest touch of "Electric Cleansing Flux Paste" (made by Buffalo Dental Mfg. Co.), on the tip; sweat solder down on the tip until there is sufficient thickness for final work, place the now partially soldered backing back on the facing, cut and bend pins slightly, wax facing into place on model. Invest, bringing the investment well up over the *soldered part* of backing; when set, pour boiling water on to the wax, cleaning it out thoroughly; paint backing with Electric Paste; dry with slow heat until model is thoroughly dry, then heat up until the crowns *glow red;* the final soldering is mere child's play, as the blowpipe flame never touches the tip at all. Using the electric paste there is absolutely no checking. Believing that borax has caused the loss of more church members than "booze." I never use it at all.—*Brief.*

Copper for Matrices.—Obtain a matrix in ordinary manner, using thin copper plate. For small cavities, fuse matrix nearly full of 18K. gold solder by holding in flame of the alcohol lamp. Replace in cavity and burnish well to cavity margins; remove and add solder until flush with margins. For large inlays, it is necessary to invest. Place a *fold of small copper wire in bottom of matrix,* and run up with gold solder, using plenty of flux; grind nearly complete before setting and, last of all, drop the inlay into nitric acid to remove the copper. The copper wire having been removed by the acid leaves a good retaining groove. Do not grind after removing from the acid until after inlay is set, as there would be danger of injuring the sharp marginal edges. These edges should be carefully burnished to the tooth while cement is soft. There are advantages in this method more patent than simply the price of the material.—V. B. Newell, *Review.*

To Enamel Aluminum.—A coating for aluminum ware, aiming to produce a coloring of durable character or in rendering the surface adapted to enameling can be obtained by a process patented in Germany, according to Metallurgie, by a Mr. Lang. The surface is covered in the first instance with a solution of a quick-silver compound—as, for instance, choloride of mercury—and by this means a coating of aluminum amalgam is obtained. After this is removed a very active process of oxidation of the surface is said to take place, which action may be interrupted by strong heating, and the aluminum oxide will serve as a foundation for the enamel. If during the process of oxidation the metal is exposed to the action of chromic acid or other suitable chromates or to some other readily reduciable substances, these compounds are at once reduced. The action of heat may also be employed to give different colored coatings, and the colors obtained may be gray, green, brown or black. They are said to resist the action of fire and render the aluminum more difficult to melt.—*Pop. Med.*

Making Over a Gold Filling Into an Inlay.—Very often there are cases presented where a large contour filling in an incisor falls out either from decay at the margins or from a faulty cavity preparation. In the former case I remove decay and reshape cavity, avoiding the margins except where decay has encroached. Burnish thin platinum 1-1000 to cavity and press filling to place. Any extensions of the cavity may be filled loosely with sponge or crystal gold, holding filling in place with a wedge. Remove all carefully by attaching a little bit of wax to a small spatula, warming same, and pressing against filling so that matrix filling and any extensions of gold may be held together. Paint cavity side of matrix with chalk and invest in tenax and plaster warm, remove wax, and flow on a little 22k solder at necessary points to unite filling to matrix, etc., or contour if necessary. Drop inlay and investment while very hot into water which will separate them promptly. Dip in nitric acid and then in soda solution. Cement to place and finish with stones, disks, etc. Does not take half the time of a gold filling and makes a better operation in a frail tooth.—*American Dental Journal.*

Method of Making Amalgam Dies.—Amalgam dies have become very popular for constructing gold and porcelain inlays, copes for crowns, and for building up teeth that have decayed far below the gum margins, with gold.

Take an impression with pink base-plate gutta percha in the following manner: Moisten the tooth and cavity with glycerine. Soften the gutta percha over an alcohol flame, being careful not to blister it and force the gutta percha into the cavity with a broad instrument. The glycerine will prevent the gutta percha from sticking in the cavity. Flow a stream of cold water over the gutta percha to harden it; carefully remove, and if the margins are perfect mix a small quantity of plaster, build it to a pyramid on a glass slab and invest the impression by forcing it down in the plaster with an instrument, being careful to have the plaster well up around the margins. When the plaster is hard mix the amalgam. I prefer to use alloy in the form of shavings, or copper amalgam is better still, as it can be used over again several times by warming it until the mercury appears in the surface and then grind it thoroughly in a mortar. Carefully pack the amalgam around the impression until it is well covered and then roll up a piece of unvulcanized rubber, about the size of a thimble, and wrap several thicknesses of rubber tape around this. Place it over the amalgam, insert in a vice and squeeze out the mercury. Leave it under pressure three or four hours, or over night. Then break away plaster, remove gutta percha, and you will find a clean cut and polished die which is easily mounted in the swage and makes a perfect matrix.—Dr. C. J. Hadley, *Dental Surgeon.*

